# Negative patch tests: what should we think about these results?^☆^

**DOI:** 10.1016/j.abd.2021.09.014

**Published:** 2022-09-06

**Authors:** Mellanie Starck, Nathalie Mie Suzuki, Mariana de Figueiredo Silva Hafner, Rosana Lazzarini

**Affiliations:** a Dermatology Clinic, Santa Casa de São Paulo, São Paulo, SP, Brazil; b Faculty of Medical Sciences, Santa Casa de São Paulo, São Paulo, SP, Brazil

Dear Editor,

Patch tests are the best tools to identify the etiological agents of allergic contact dermatitis (ACD). They are indicated in suspected ACD, in chronic eczemas with no defined etiology, in occupational contact dermatitis, and for the investigation of drug reactions with a delayed hypersensitivity mechanism.^1^ Responses to patch tests are evaluated using morphological criteria already described by the International Contact Dermatitis Research Group (ICDRG). A negative result in a patient with some type of eczema can be frustrating; thus, it is important to know the differential clinical diagnoses and the reasons why a test can be negative.

The present study aims to determine the frequency of negative patch tests in patients with clinical suspicion of ACD, their epidemiological profile and final diagnoses. This study was approved by the Human Research Ethics Committee under number 20285919.1.0000.5479.

This is a descriptive retrospective study, carried out with the analysis of medical records of patients with ACD from 2013 to 2018 who had negative results or irritative reactions to the tested substances. The patch test series used were selected according to the suspected diagnosis: Brazilian Standard, Cosmetics and Corticosteroids (FDA Allergenic/Rio de Janeiro, Brazil); Latin American, Expanded Series Patch Testing, Phototest, Footwear, Metals, Ulcers (Chemotechnique Diagnostics/Vellinge, Sweden); Hair, Nails, and Anti-Inflammatories (IPI-ASAC/São Paulo, Brazil). The tests were applied to the patients upper back region using AlergoChamber™ (Neoflex/Sertãozinho, São Paulo, Brazil). The obtained data were tabulated and analysed.

Of the 694 patients submitted to patch tests with a diagnostic hypothesis of ACD, 116 (16.7%) were negative for all tested substances, 72 of which (62.1%) were female. Age ranged from 10 to 89 years, with a mean age of 47.6 years. The anatomic areas are shown in Table 1.

**Table 1:** Distribution of patients with negative patch tests according to the site of the dermatosis.

Affected body area	n	%
Hands	48	18.8
Arms	29	11.4
Forearms	25	9.8
Legs	25	9.8
Feet	25	9.8
Trunk	23	9.0
Cervical region	19	7.4
Thigh	14	5.5
Face	14	5.5
Eyelids	11	4.3
Scalp	7	2.7
Pelvis	6	2.3
Axilla	5	2.0
Generalized	4	1.6
Total	255^a^	100

Data: Dermatology Clinic, Irmandade da Santa Casa de Misericórdia de São Paulo, 2013‒2018.

a Some patients had more than one affected location.

It is noteworthy that 12 patients (1.73%) were using immunosuppressants, such as methotrexate, corticosteroids, cyclosporine, and azathioprine for at least six months. Of these, three patients (25%) tested negative for all substances.

The final diagnoses of patients with negative patch tests (Table 2) were 41 (34.2%) cases with irritant contact dermatitis (ICD), 21 (17.5%) with atopic dermatitis (AD), and 7 (5.9%) with psoriasis. Some non-eczematous diagnoses were also identified, such as contact urticaria in three (2.5%), erythema multiforme, and vitiligo in one case each (0.8%). The diagnoses were not completed in 20 patients (16.7%), who were lost to follow-up or showed spontaneous improvement of the lesions.

**Table 2:** Distribution of patients with negative patch tests according to the final diagnosis.

Final diagnosis	n	%
Irritant contact dermatitis	41	34.2
Atopic dermatitis	21	17.5
Psoriasis	7	5.8
Lichen simplex chronicus	4	3.3
Seborrheic dermatitis	3	2.5
Contact urticaria	3	2.5
Drug reaction	3	2.5
Phototoxic contact dermatitis	2	1.7
Nummular eczema	2	1.7
Stasis dermatitis	2	1.7
True dyshidrosis	2	1.7
Pruritus	2	1.7
Perioral dermatitis	1	0.8
Mycosis fungoides	1	0.8
Erythema multiforme	1	0.8
Lichen amyloidosus	1	0.8
Sensitive skin syndrome	1	0.8
Irritant reaction of the upper airways	1	0.8
Vitiligo	1	0.8
Verruca plana	1	0.8
Undefined	20	16.7
Total	120^a^	100

Data: Dermatology Clinic, Irmandade da Santa Casa de Misericórdia de São Paulo, 2013‒2018.

a Some patients had more than one diagnosis.

In the present study, ICD was the main final diagnosis, as this is the most common of contact dermatitis and its confirmation is made through clinical aspects in addition to negative patch tests. In some situations, the test is performed to exclude differential diagnoses and also to verify the coexistence of the diagnosis of ACD with other dermatoses, such as AD and psoriasis, which are also found among the final diagnoses established.

In the presence of negative results, it is still valid to consider failure in the technique, such as lack of adhesion of the test chambers and the final reading performed within a shorter time interval than the recommended one, in addition to the unavailability of the suspected allergen. Questions related to the commercial product used to perform the testing can have a great influence on the response since the concentration of the allergen, the vehicle and the ability of the allergen to penetrate the skin depend on the manufacturer quality standards.

The immunosuppressive effect of medications and sun exposure prior to testing can also result in false negative tests. In the present study, a higher percentage of negative tests was observed in patients using immunosuppressants, when compared to the general group, but more studies in this field are needed to understand these results.

It was observed that 16.7% of the patients tested had negative results, which is a lower rate than that observed in the world literature. The patients profile is in agreement with that in the literature, with the hand being the body part most often affected by the dermatosis (18.8%) and the most frequent final diagnosis being ICD, with 34.2%; similar to what was found in the literature.^2^

In a retrospective cross-sectional study by Warshaw et al., of 34,822 patients tested by the North American Contact Dermatitis Group (NACDG), almost one-third (31.3%) had negative patch tests.^2^ Studies documented by the European Surveillance System on Contact Allergy (ESSCA) show that the percentages of negative patch tests for the European standard series tested by the ESSCA and for the specific substances added by country were: 60% in Denmark, 57% in the UK, 54% in Italy, 46% in Austria.^3^

National studies regarding allergen positivity from 2003 to 2010, considering the Brazilian standard tests, report that 59.2% to 64.68% of patients had a positive test result for at least one substance.^4^, ^5^

In the present study, most of the assessed patients underwent not only the standard Brazilian tests but also additional series. This may have contributed to a lower percentage of negative results in our findings when compared to the literature, in which test data using only the standard series is used as the reference. After a thorough anamnesis, the outpatients are submitted to additional tests to guide the investigation of the disease etiology.

The performance of patch tests when there is clinical suspicion helps in the diagnosis of the etiology in most cases. In the presence of a negative test result, other causes of eczema should be considered; mainly ICD and AD, in addition to the less frequent non-eczematous differential diagnoses. Another relevant point to consider is the possibility of not having tested the substance causing the ACD. In the present study, most patients underwent additional testing, not just the Brazilian standard series, which contributed to a lower percentage of negative results when compared to the literature.

## Financial support

None declared.

## Authors’ contributions

Mellanie Starck: Collection, analysis and interpretation of data; critical review of the literature.

Nathalie Mie Suzuki: Drafting and editing of the manuscript; critical review of the manuscript.

Mariana de Figueiredo Silva Hafner: Design and planning of the study; drafting and editing of the manuscript; critical review of the manuscript.

Rosana Lazzarini: Approval of the final version of the manuscript; design and planning of the study; effective participation in research orientation.

## Conflicts of interest

None declared.
